# Self-care and self-management in diabetes: concepts, theories and practices

**DOI:** 10.1007/s11096-025-01941-z

**Published:** 2025-06-09

**Authors:** Saranya Puzhakkal, Sallianne Kavanagh, Barbara Conway, Chia Siang Kow, Syed Shahzad Hasan

**Affiliations:** 1 Department of Pharmacy, School of Applied Sciences, University of Huddersfield, Huddersfield, HD1 3DH UK; 2 Sheffield Teaching Hospitals NHS Foundation Trust, Sheffield, UK; 3 Department of Pharmacy Practice, School of Pharmacy, IMU University, Kuala Lumpur, Malaysia

**Keywords:** Diabetes, Self-care, Self-management, Type 2 diabetes

## Abstract

Diabetes mellitus is a progressive disorder that requires the active participation of individuals with diabetes to improve management and delay the onset of acute and chronic complications. Effective diabetes care necessitates a multifaceted approach involving insulin therapy, non-insulin anti-diabetic drugs and lifestyle adjustments. Self-management and self-care are integral, yet distinct, components of diabetes care. While both play a pivotal role in optimising diabetes care, their distinctions and similarities are often misunderstood. These terms are frequently used interchangeably. Although some activities are unique to each concept, inconsistencies exist in the published literature. Self-management refers to patient behaviours guided by healthcare professionals, whereas self-care encompasses autonomous actions patients take to maintain health and prevent complications. Both concepts involve activities that foster long-term collaboration between patients and healthcare professionals.

## Background

Diabetes mellitus, a chronic condition characterised by impaired glucose metabolism, is a significant global health concern [1]. According to the most recent International Diabetes Federation (IDF) Diabetes Atlas (2025), 11.1% of adults aged 20 to 79, approximately 1 in 9, have diabetes, with more than 40% being unaware of the condition. The IDF report also estimates that by 2059, 853 million people (1 in 8 adults) will have diabetes, representing a 46% increase [2]. Type 2 diabetes is the most common form, resulting from inadequate insulin production and insulin resistance. Without proper management, diabetes leads to complications such as cardiovascular disease, neuropathy, retinopathy, and chronic kidney disease, all of which impose a substantial physical, emotional, and economic burden on patients and healthcare systems [3].

Effective diabetes management requires a comprehensive approach involving pharmacotherapy, lifestyle adjustments, and patient engagement [4]. Self-management and self-care practices require active participation from patients and empower individuals to take charge of their health, improve outcomes, and reduce complications [5]. However, the interchangeable use of self-management and self-care terminology in research and practice creates confusion, often obscuring their unique and individual contribution to diabetes care. This article aims to clarify distinctions between these concepts, emphasise their interdependence, and advocate for holistic and personalised strategies in managing diabetes.

## Theoretical basis for self-care or management

Self-care or self-management is theoretically linked to social cognitive theory and behaviour change models. It draws on theories focused on health maintenance and autonomy-focused frameworks, involving goal setting and collaborative work with healthcare professionals [6–13].

The concepts surrounding diabetes self-care or self-management are shaped by behavioural and psychological theories (as shown in Table 1). These theories or frameworks describe how individuals with chronic conditions (e.g., diabetes) adopt, maintain, and adjust behaviours to improve health outcomes. They provide a foundation for both individuals and healthcare professionals, emphasising patient empowerment, self-efficacy, and the active role of patients in managing their health. A prominent example is Orem’s Self-Care Deficit Nursing Theory (SCDNT), which identifies self-care deficits, guides intervention planning, and empowers patients to improve their self-care abilities [6].

**Table 1:** A summary of key theories describing the concept of self-care in chronic disease (e.g., diabetes)

Source	Theory/framework	Description
Deci EL, Ryan RM, 1985 [12]	Self-determination theory (SDT)	This emphasises the importance of intrinsic motivation and psychological needs, such as autonomy, competence, and relatedness, in fostering sustained health behaviour change
Bandura A, 1986 [7]	Social cognitive theory (Bandura)	This focuses on self-efficacy, which is the belief in one’s ability to perform specific behaviours such as successfully managing blood glucose levels, adhering to medication regimens, and engaging in effective self-management
Rosenstock IM, Strecher VJ, Becker MH, 1988 [9]	Health belief model (HBM)	This approach focuses on individuals’ perceptions about their susceptibility to complications, the seriousness of diabetes, the benefits of taking action, and the barriers to action that impact their engagement in self-care behaviours. It also focuses on cue-to-action and self-efficacy
		This concept was early developed by Hochbaum, GM, 1958 [8]
Ajzen I, 1991 [11]	Theory of planned behaviour (TPB)	This approach focuses on the impact of individuals’ intention to perform a behaviour (e.g., glucose monitoring, physical activity), patients’ attitudes toward the behaviour, social pressures, and perceived control over or self-efficacy in the behaviour
Wagner EH, Austin BT, Von Korff M, 1996 [10]	Chronic care model (CCM)	This model discusses shifting chronic disease management, such as diabetes management, from an acute, healthcare provider-driven model to proactive, patient-centred, team-based care
Orem DE, 2001 [6]	Orem’s self-care deficit nursing theory (SCDNT)	This highlights individuals’ responsibility to care for themselves (e.g., actively monitor blood glucose, administer medication, and manage diet). Nurse support is available when people cannot meet their self-care needs
Riegel B, Jaarsma T, Strömberg A, 2012 [13]	Middle-range theory of self-care of chronic illness (MRTSC)	This theory highlights maintaining health through health-promoting practices in managing a chronic illness, focusing on self-care maintenance, monitoring, and self-care management

## Definitions and distinctions

To summarise the distinctions and similarities, a search was conducted in Medline, CINAHL, and PsycINFO to explore relevant articles and documents related to diabetes self-care and self-management. This included various interchangeable terms such as ‘diabetes self-care,’ ‘diabetes self-management education,’ ‘diabetic patient-centred care,’ and ‘diabetes customised care.’ This broad approach ensured the comprehensive extraction of diverse perspectives and concepts. The literature search identified 15 relevant documents, selected based on their relevance to diabetes self-care or self-management and their contribution to understanding the intricacies of diabetes care.

Table 2 summarises the nuanced distinctions and commonalities between these concepts. It highlights the varied interpretations of self-management and self-care, emphasising their complementary roles in comprehensive diabetes management [14–29]. Organisations such as the National Institute for Health and Care Excellence (NICE) and the American Diabetes Association (ADA) emphasise the importance of educating patients, their family members, and caregivers to ensure they possess the knowledge, skills, and abilities necessary for effective diabetes self-care [15, 24]. According to the ADA, diabetes self-management and self-care are closely connected processes that empower individuals to manage their condition actively, ultimately leading to improved health outcomes and quality of life [23].

**Table 2:** Summary of self-management and self-care concepts in diabetes care

Source	Term used	Description
Al-Dwaikat TN, Ali AM, Khatatbeh H, 2023 [14]	Self-management social support	A supportive social network exhibiting supportive and reinforcing behaviours that could facilitate positive behavioural change and promote disease self-management, improving patient behavioural and psychosocial outcomes
National Institute for Health and Care Excellence (NICE), 2022 [15]	Structured education programme for adults with type 2 diabetes	NICE does not provide a singular, formal definition but recommends that any structured diabetes education programme for adults with type 2 diabetes must be evidence-based and tailored to individuals’ needs
		It emphasises supporting the person, family members, and carers in developing attitudes, beliefs, knowledge, and skills to effectively self-manage diabetes
Wilson V, 2021 [16]	Self-care in diabetes	It involves developing knowledge and awareness by learning to live with the complex nature of the condition in a social context
		Structured education and psychological support are the key enablers to living well with diabetes
Powers MA, Bardsley J, Cypress M, et al., 2021 [17]	Diabetes self-management	DSM targets maintaining individualised goals for glycemic control through comprehensive lifestyle behaviours, including dietary management, physical activity, weight management, optimising medication-taking behaviours, and glucose self-monitoring
Lee J, Lee EH, Chae D, et al., 2020 [18]	Diabetes self-management	Diabetes self-management is a multidimensional construct that includes diet, exercise, medications, self-monitoring of blood glucose, foot care, and less common domains such as psychological coping
		Self-care refers to regularly performed behaviours planned in collaboration with healthcare professionals to achieve optimal health outcomes in daily living. These behaviours comprise multiple components, such as diet, physical activity, self-monitoring of blood glucose, medication adherence, and coping with emotional distress
De Man J, Aweko J, Daivadanam M, et al., 2019 [19]	Self–management	A key component for adequate prevention and treatment of type 2 diabetes and other non-communicable diseases is that it improves health outcomes through better treatment adherence
Adu MD, Malabu UH, Malau-Aduli AE, et al., 2019 [20]	Diabetes self-management	Day-to-day activities or actions a person must undertake to control or reduce the impact of disease on their health and well-being, to prevent further illness. These actions involve engaging in recommended behavioural activities such as medication adherence, being active, monitoring, reducing risks, problem-solving, and healthy coping, all necessary to manage diabetes successfully
Eva JJ, Kassab YW, Neoh CF, et al., 2018 [21]	Self-care	It incorporates deliberate moves to look after physical, mental, and emotional health. The best characteristics of self-management are the patient’s decision and behaviour when they engage in any chronic disease that affects their well-being. Self-care practices involve various areas, including food, exercise, medicine, emotion, sleep, and medical care
Martz E, 2017 [22]	Self-management	It refers to an individual’s ability to manage the clinical and psychosocial consequences and lifestyle changes inherent in living with a chronic condition
American Diabetes Association ADA, 2017 [23, 24]	Diabetes self-management education and support (DSMES)	No single formal definition of self-management or self-care
		It emphasises diabetes self-management education and support, an ongoing process that facilitates the knowledge, skills, and abilities necessary for self-care
		It describes self-care as individuals’ daily activities and decisions to manage their diabetes effectively, including monitoring blood glucose levels, adhering to medication regimens, maintaining a healthy diet, engaging in regular physical activity, and coping with the psychosocial aspects of living with diabetes
Rural Health Information (RHI) Hub, 2012 [25]	Diabetes self-management	It refers to the individual’s activities and behaviours to control and treat their condition. People with diabetes must monitor their health regularly
		Diabetes self-management typically occurs in the home and includes testing blood sugar (glucose), consuming balanced meals and appropriate portion sizes, engaging in regular exercise, drinking water and avoiding dehydration, taking medications as prescribed, adjusting medications as needed, conducting self-foot checks, and monitoring other signs or symptoms caused by diabetes
Diabetes UK, 2009 [26]	Self-management and self-care	This defines self-care is something everyone does every day, but it may be more difficult for someone with a long-term condition
		Self-management is how a person develops the skills to manage their condition
		Health and social care providers are responsible for supporting self-management and self-care, but unpaid carers often provide it
		It focuses on managing the relationships between food, activity, and medications. It targets goals tailored to individual needs, such as foot care, weight loss, injection technique, self-monitoring activities, managing acute complications such as hypoglycaemia and hyperglycaemia, and understanding legislative issues related to employment and driving
Koch T, Jenkin P, Kralik D, 2004 [27]	Self-management	Healthcare providers should embrace fresh perspectives on managing chronic illness to help people develop self-agency. Respecting an individual’s expertise in managing their disease improves this process
Lorig KR, 2003 [28]	Self-management skills	Skills required for self-management: 1) problem-solving, 2) decision-making, 3) resource utilisation, 4) the formation of a patient-provider partnership, 5) action planning and behaviour change, and 6) patient tailoring management plans to suit their needs
Corbin JM, Strauss A, 1988 [29]	Self-management	It comprises three distinct sets of activities: (1) medical management, e.g. taking medication and adhering to dietary advice; (2) behavioural management, e.g. adopting new behaviours in the context of chronic disease; and (3) emotional management, e.g. dealing with the feelings of frustration, fright, and despair associated with chronic disease

DSM, diabetes self-management

Self-management and self-care are integral, yet distinct, components of diabetes care. Self-management involves patient behaviours guided by healthcare professionals, including goal setting, behaviour modification, and collaborative action planning. For instance, diabetes self-management education (DSME) programs aim to provide patients with the tools and knowledge necessary for effective disease control. These programs emphasise partnership between patients and providers, fostering shared responsibility for achieving glycaemic targets and preventing complications.

In contrast, self-care refers to patients’ autonomous actions to maintain health and prevent complications. These include adhering to prescribed diets, engaging in regular physical activity, and monitoring blood glucose levels. Self-care reflects the patient’s daily experiences and choices in managing their condition. Unlike self-management, which often involves structured professional input, self-care highlights the patient’s capacity for independent decision-making.

The distinction between self-care and self-management is not merely semantic; it has essential theoretical and practical implications. From a clinical pharmacy perspective, understanding whether an activity falls under self-care or self-management influences the type of intervention required, whether autonomous support tools (e.g., glucose diaries) or structured programs (e.g., diabetes self-management education, or DSME) are more appropriate. For policymakers and service designers, these distinctions inform service delivery models and clarify whether responsibilities lie with health systems or individuals.

The concepts of self-care and self-management adopted in this article are grounded in existing literature specific to diabetes care. While both concepts are applied across various chronic conditions, their operationalisation in diabetes involves unique behaviours such as blood glucose monitoring, carbohydrate counting, insulin titration, and foot care [4, 17]. These activities in diabetes care may differ in emphasis or nature compared to those in conditions such as asthma or hypertension. Therefore, the information presented here reflects the nuances of diabetes-specific self-care and self-management practices described in previous conceptual frameworks [18, 28, 29].

## Activities and shared concepts

Diabetes self-management and self-care encompass many activities, several of which overlap. Key activities include blood glucose monitoring, medication adherence, lifestyle modifications, and emotional support. Blood glucose monitoring is crucial in diabetes management, as it helps patients identify fluctuations and respond promptly to prevent acute complications, such as hypoglycaemia or hyperglycaemia. Medication adherence, including non-insulin anti diabetic drugs, insulin, or other injectables, is essential for maintaining glycaemic stability.

Lifestyle modifications, such as adopting a balanced diet and engaging in regular physical activity, are critical in reducing the risk of complications and improving quality of life [14, 16, 29]. Emotional support is equally vital, as patients often experience stress, anxiety, or depression related to their condition. Addressing these psychological challenges is integral to achieving long-term management success. For example, while self-care emphasises autonomy in meal planning and exercise routines, self-management incorporates structured education and goal setting. Activities that improved knowledge, skills, and ability and emotional and psychological support were most frequently cited in the literature, as summarised in Table 1.

**Fig. 1:**
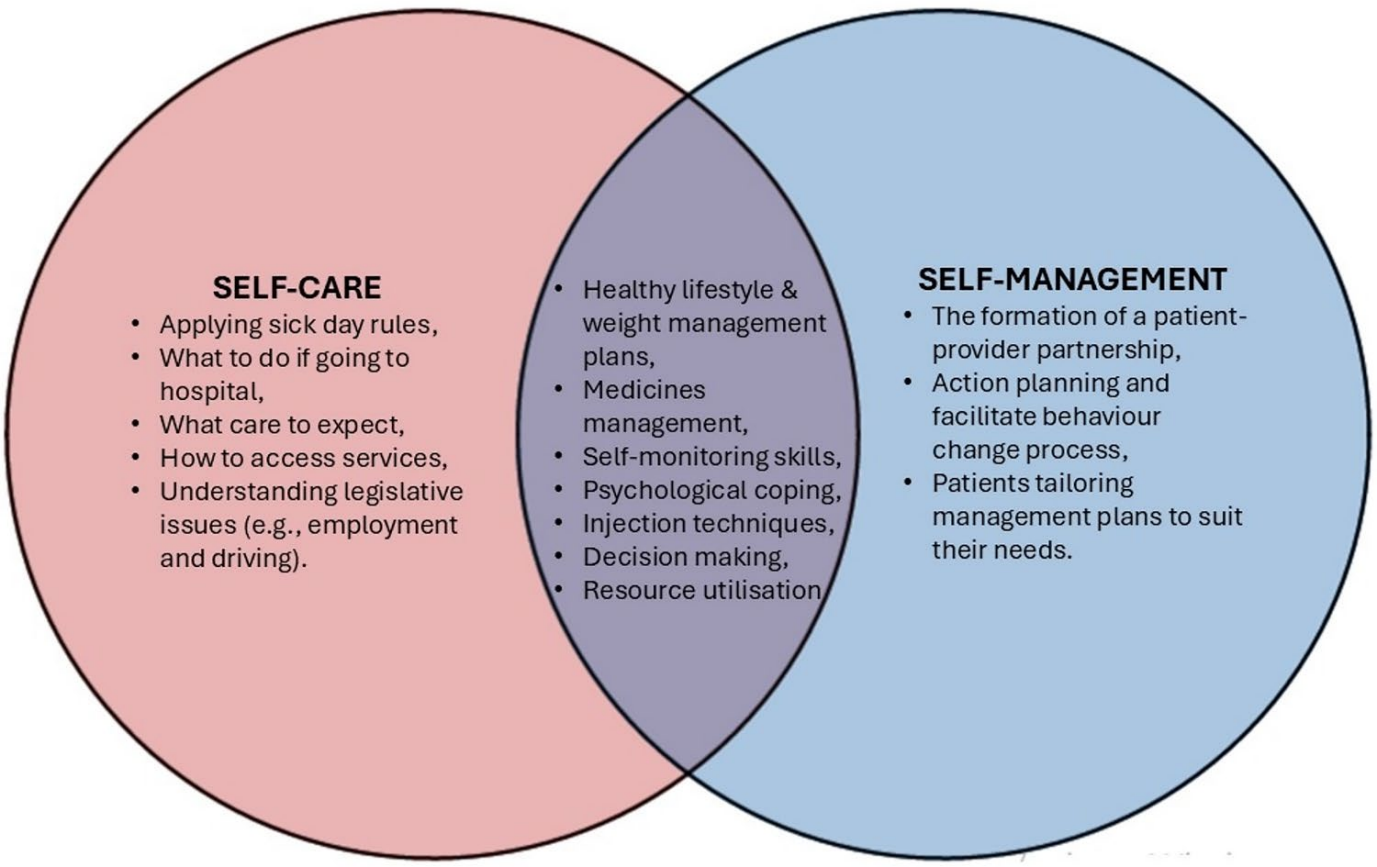
Diagrammatic illustration of the distinctions and similarities between self-management and self-care (based on [14–29])

Figure 1 illustrates the distinctions and similarities between self-care and self-management, as outlined by the sources in Table 1. It demonstrates how self-management incorporates input from healthcare providers, while patient-driven behaviours and actions primarily guide self-care. Together, these approaches form a synergistic and complementary framework that addresses the clinical and psychosocial aspects (or dimensions) of diabetes care.

## Holistic and personalised approaches

In addition to a well-established theoretical framework and a clear definition, effective (and successful) self-care or management requires a holistic approach tailored to individual needs, preferences, and circumstances [30]. Cultural background, socioeconomic status, and health literacy significantly influence a patient’s ability to engage in self-care and self-management. For instance, patients from diverse cultural backgrounds may face barriers related to dietary habits, language, or traditional beliefs about health. Addressing these barriers through culturally tailored education programs can improve accessibility and engagement.

Technology plays an increasingly important role in supporting personalised diabetes care. Mobile health apps, wearable devices, and telemedicine platforms enable patients to monitor blood glucose levels, track physical activity, and receive real-time feedback from healthcare providers. These tools not only enhance patient engagement but also facilitate continuous care, particularly for those in remote or underserved areas.

Emotional well-being is another critical aspect of holistic care. Living with diabetes often involves coping with emotional distress, which can negatively affect adherence and quality of life [31]. Interventions such as counselling, peer support groups, and stress management programs can help patients build resilience and maintain motivation for long-term management.

## Challenges and barriers

Despite the benefits of self-management and self-care, several barriers hinder their implementation. Limited resource access is a significant challenge, particularly in rural or low-income areas. Patients in these settings may lack access to educational programs, healthcare providers, or essential medical supplies such as glucose meters and test strips.

Low health literacy also poses a barrier, as patients may struggle to understand complex medical instructions or make informed decisions about their care. This is particularly concerning for populations with limited educational opportunities or language proficiency. In addition, psychosocial factors such as stigma, depression, and lack of social support can diminish motivation and hinder participation in self-care or structured self-management programs.

Incorporating real-world experiences from patients and healthcare professionals can enrich our understanding of how self-care and self-management are implemented. For instance, some patients may view self-care as intuitive daily maintenance, while professionals may see it as an extension of structured care [32]. Practitioners often report challenges in engaging patients expected to transition from structured education into autonomous self-care, especially without follow-up support. Including these perspectives in future research could guide the development of contextually relevant and patient-centred interventions.

Innovative strategies are needed to overcome these barriers. Simplified educational materials, community-based interventions, and supportive policies can promote equitable care and empower patients to take control of their health. For example, community health workers trained in diabetes education can provide culturally relevant support, bridging gaps in access and understanding.

## Implications for practice and research

Integrating self-management and self-care into routine diabetes care requires a collaborative approach from people with diabetes, healthcare providers and researchers, alongside a practical theoretical framework. Healthcare providers must be trained to deliver personalised, patient-centred care that addresses the diverse needs of their patients. This includes developing communication skills, fostering trust, and using motivational interviewing techniques to enhance patient engagement. This framework can address similar issues, such as self-administration and self-management of insulin. Self-administration refers to taking insulin as prescribed by a doctor, whereas self-management refers to selecting and administering insulin doses based on self-measured blood glucose levels [33].

The differentiation between self-care and self-management has implications for commissioning diabetes services. In many healthcare systems, commissioned services tend to focus on structured interventions such as DSME or personalised care plans, which fall under self-management [34]. Self-care, being patient-initiated and often informal, may be overlooked in funding structures. Recognising this distinction can guide policymakers and commissioners in designing services that bridge both domains, ensuring that support for autonomous self-care is also embedded into formal care pathways.

While the distinctions between self-care and self-management are conceptually valuable, they are not consistently represented in international guidelines. For example, the American Diabetes Association (ADA) recognises structured diabetes self-management education and support (DSMES) as essential but does not always delineate its boundaries with self-care practices [35]. Similarly, NICE and WHO documents include both terms but with varying interpretations [36, 37]. A more explicit application of these terms within policy documents could enhance consistency in implementation across healthcare systems.

Research should focus on evaluating the effectiveness of innovative interventions, such as artificial intelligence-based decision support systems and remote monitoring technologies. These tools can transform diabetes care by providing real-time insights and enabling personalised recommendations. Additionally, studies should explore the scalability of successful models to ensure they are accessible to diverse populations.

## Conclusion

Self-management and self-care are integral to effective diabetes management. While these concepts share many activities, their distinctions lie in the degree of autonomy and professional involvement they require. Together, they provide a comprehensive framework for empowering patients, addressing barriers, and fostering collaboration between patients and healthcare providers.

Future efforts should prioritise holistic, personalised approaches that leverage technology, address individual barriers, and promote equitable access to care. By integrating self-management and self-care into routine practice, healthcare systems can improve outcomes, enhance quality of life, and reduce the burden of diabetes on individuals and society.
